# Ultrasound Imaging and Guidance for Cervical Myofascial Pain: A Narrative Review

**DOI:** 10.3390/ijerph20053838

**Published:** 2023-02-21

**Authors:** Vincenzo Ricci, Kamal Mezian, Ke-Vin Chang, Domiziano Tarantino, Orhan Güvener, Fabrizio Gervasoni, Ondřej Naňka, Levent Özçakar

**Affiliations:** 1 Physical and Rehabilitation Medicine Unit, Luigi Sacco University Hospital, ASST Fatebenefratelli-Sacco, 20157 Milan, Italy; 2 Department of Rehabilitation Medicine, First Faculty of Medicine and General University Hospital, Charles University, 12800 Prague, Czech Republic; 3 Department of Physical Medicine and Rehabilitation, National Taiwan University Hospital, Bei Hu Branch, Taipei 10845, Taiwan; 4 Department of Public Health, Rehabilitation Unit, University Federico II of Naples, 80131 Naples, Italy; 5 Department of Physical and Rehabilitation Medicine, Mersin University Medical School, 33000 Mersin, Turkey; 6 Institute of Anatomy, First Faculty of Medicine, Charles University, 12800 Prague, Czech Republic; 7 Department of Physical and Rehabilitation Medicine, Hacettepe University Medical School, 06100 Ankara, Turkey

**Keywords:** trigger point, cervical spine, muscle, sonography, intervention

## Abstract

Cervical myofascial pain is a very common clinical condition in the daily practice of musculoskeletal physicians. Physical examination is currently the cornerstone for evaluating the cervical muscles and identifying the eventual presence of myofascial trigger points. Herein, the role of ultrasound assessment in precisely localizing them is progressively mounting in the pertinent literature. Moreover, using ultrasound, not only the muscle tissue but also the fascial and neural elements can be accurately located/evaluated. Indeed, several potential pain generators, in addition to paraspinal muscles, can be involved in the clinical scenario of cervical myofascial pain syndrome. In this article, the authors extensively reviewed the sonographic approach for cervical myofascial pain in order to better diagnose or guide different procedures that can be performed in the clinical practice of musculoskeletal physicians.

## 1. Introduction

Chronic non-specific neck pain is one of the most frequent reasons for medical consultation with primary care physicians and pain clinics [1,2]. It is a well-recognized socioeconomic problem and a frequent cause of job absenteeism as well [3]. Under this umbrella definition, cervical myofascial pain syndrome (MPS) is, by far, the most prevalent condition. It constitutes more than 90% of cases and presents with peculiar symptoms and signs [3]. Restriction of the active/passive range of motions of the cervical spine together with local/referred pain represents the most common clinical feature [1,2]. Myofascial trigger points (MTrPs), commonly described as palpable hard nodules housed inside taut bands of skeletal muscles, are considered the hallmarks of MPS [4]. In daily practice, X-ray examination is usually considered the first-line diagnostic modality to assess cervical spine morphology. It is also used to exclude red flags, such as unstable vertebral fractures and bone tumors. Currently, the diagnosis of cervical MPS is mainly based on clinical examination through manual identification of the MTrPs within target muscles, as well as prompt reproduction of local/referred cervical pain induced by palpation [4,5]. Moreover, the local twitch response of the MTrPs and release of the taut-band tension induced by manual maneuvers or dry needling are considered additional clinical findings that need more experience to be correctly identified [1,4,5]. Taking into account the high variability of the manual identification of MTrPs in daily practice between different operators [6], several authors have proposed ultrasound (US) imaging to optimize the diagnosis and management of myofascial pain [7,8]. In this sense, several sonographic features of MTrPs have been described both in B-mode and color/power Doppler imaging. A hypoechoic nodule with clearly defined edges and heterogeneous internal echotexture is the classic sonographic aspect of the trigger point [7,8,9]. Furthermore, not only the better identification of MTrPs [9] but also safe guidance during their interventions [10,11] can be guaranteed with US examination. Lastly, several fascial structures and neural elements can precisely be identified and injected under US guidance at the level of the cervical spine for comprehensive management of the complex clinical scenario of MPS.

The main purpose of this paper is to extensively review the scientific literature pertinent to both US assessment of cervical myofascial pain and US-guided procedures. The authors tried to propose an easy-to-use guide for the clinical practice of musculoskeletal physicians.

## 2. Materials and Methods

In order to develop a practical, ready-to-use guide concerning the US-guided procedures used for cervical myofascial pain, the authors planned a three-step workflow as described below:

1st Phase: An extensive review of the scientific literature related to the anatomical, histological, biochemical, and sonographic features of MTrPs was performed. Of note, correct identification of MTrPs using US requires a comprehensive knowledge of their histo-anatomical features.

2nd Phase: Focusing on the cervical MPS, the review was expanded to (i) pain generators different from the MTrPs e.g., fascial/neural structures and, (ii) US-guided interventions commonly described in the literature. The complex interaction between several tissues (as pain generators) at the level of the cervical spine is a real challenge to tackle for musculoskeletal physicians.

3rd Phase: Based on the aforementioned phases of the workflow, a “pain generator-based approach” for US-guided procedures in cervical MPS was proposed.

## 3. Results and Discussion

Considering the nature of the present review, the authors combined the results and discussion sections in order to provide a step-by-step approach to cervical MPS, as follows:

- MTrP complex
- Sonographic assessment of the MTrPs
- Fascial structures and neural elements
- US-guided procedures

For each and every pain generator potentially involved in cervical MPS, the corresponding interventional technique was described in order to optimize the practical management of patients in daily practice (Table 1).

### 3.1. Myofascial Trigger Point Complex

The painful area in MPS is usually characterized by the presence of multiple elements anatomically and functionally connected to each other, i.e., collectively known as the MTrP complex [1,2]. The taut band of the skeletal muscle presents a leading contraction nodule in the central portion, and peripheral trigger points within the proximal and distal attachment sites to the bone and/or periosteum [5]. Within the major trigger point, a variable mixture of physiological and pathological muscle fibers can be identified. The former contains sarcomeres of normal length, whereas the latter shows a mixture of shortened and elongated sarcomeres producing contraction knots and mechanical deformation of the extracellular matrix [12,13].

Structural changes of the muscle fibers and endomysium are both considered the main histopathological features leading to macroscopic abnormalities of the muscle echotexture with the development of well-known hypoechoic nodules. Some authors have also demonstrated the use of magnetic resonance imaging whereby focal edematous changes (T2 hyperintensity signals) related to the local presence of abnormal fluids inside the micro-cracks of the intercellular scaffold of the skeletal muscle (i.e., intercellular edema) can be detected [14,15]. Interestingly, increased concentrations of glycosaminoglycans within the pathological gaps of the endomysium are very prone to retaining fluids around the contraction knots of the muscle fibers. They counteract the washout of nociceptive substances, proinflammatory molecules, and protons [16]. The aforementioned biochemical milieu seems to sensitize the muscle nociceptors, leading to muscle hyperalgesia.

The clinical effects of massage could be partially related to the squeezing/drainage of extra water bound to the glycosaminoglycans within the intercellular matrix of the painful muscle area [16]. The authors speculate that a similar release effect of the trigger point could be achieved by performing US-guided dry needling with multiple back-and-forward movements of the needle using a fan-like technique.

### 3.2. Sonographic Assessment of MTrPs

**Figure 1:**
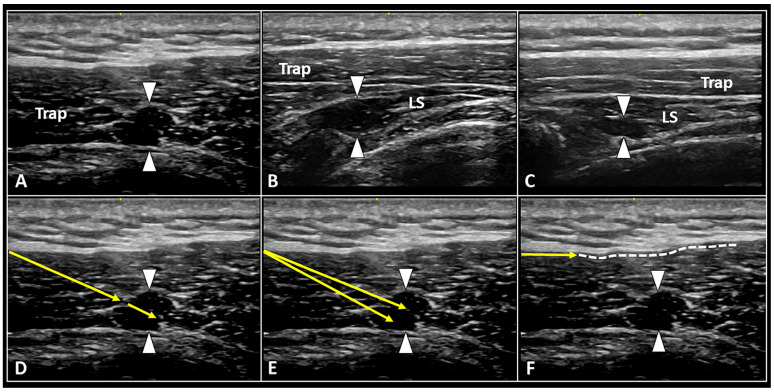
Ultrasound images show MTrPs (white arrowheads) at the level of cervical spine located both within superficial muscles (**A**), e.g., upper fibers of trapezius (Trap), and deeper muscle planes (**B**,**C**), e.g., the levator scapulae (LS). Under real-time US guidance, back-and-forward movements of the needle (yellow arrow) to ‘release’ the nodule (**D**), fan-like dry needling of the trigger point (**E**), and hydro-dissection (**F**) of the histological interface between the muscle and the deep fascia (white dotted line) can be performed in a single multi-step procedure.

The typical pattern of MTrPs during US assessment is characterized by a focal hypoechoic area with heterogeneous internal texture [7,8]. Its morphology is highly variable; among many, spherical, elliptical, and band-like shapes are the most commonly encountered in daily practice (Figure 1) [11].

Considering the complex anatomical architecture of the muscles and fasciae of the cervical spine, a step-by-step sonographic approach is suggested to optimize the evaluation of patients with myofascial pain. Herein, the authors suggest starting with a wide sonographic scan of the painful area. It is noteworthy to acquire a panoramic view of both the superficial and deep muscle layers. This pre-procedural phase can be very helpful, especially in patients in whom identification of MTrPs is otherwise difficult (only with palpation), either due to concomitant/diffuse cervical muscle spasm or their deep localization. Likewise, especially for beginners, a slow shift of the probe over the painful area is recommended to accurately depict minor changes in the muscle echotexture.

In doubtful cases, the probe can be gently tilted to avoid fake hypoechogenicity of the muscle tissue related to the anisotropy artifact [9,17]. Indeed, muscle tissue is more prone to the aforementioned acoustic artifact due to the presence of multiple muscle fascicles with different spatial orientations [18]. In some cases, tiny septae of connective tissue widely ramified within the muscle can make the peripheral boundary of the MTrP cloudy. They are also hardly detectable with US. The authors suggest adopting an oscillatory technique with very small inclinations of the probe over the hypoechoic nodule to optimize its sonographic visibility and to reduce the “disturbing effect” of the connective ramifications [9].

**Figure 2:**
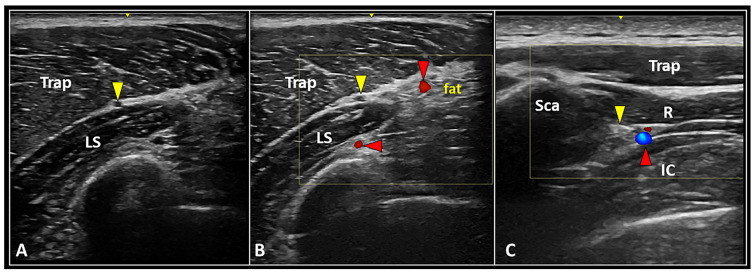
Longitudinal US scan (**A**) clearly shows the anatomical location of the spinal accessory nerve (yellow arrowhead) within the interfascial space between the trapezius (Trap) and levator scapulae (LS) muscles. Accurately setting the color Doppler box, superficial and deep branches of the transverse cervical artery (red arrowheads) can be promptly identified to plan a safe intervention (**B**). Transverse ultrasound scan with color Doppler imaging (**C**) shows the dorsal scapular nerve (yellow arrowhead) and artery (red arrowhead) running inside the interfascial plane between the rhomboid (R) and intercostalis (IC) muscles medial to the medial edge of the scapula (Sca).

Second, B-mode evaluation should be coupled with power Doppler imaging for each and every patient in clinical practice. Not simply the intra/peri-nodular perfusion pattern, but also the eventual location of large neurovascular elements surrounding the MTrP, can be promptly assessed (Figure 2) [11]. The latter is paramount to avoid unintentional collateral injury—in other words, to plan for an accurate and safe procedure.

Lastly, the authors suggest always performing precise sono-palpation of the hypoechoic nodule to verify if the local or referred pain of the patient can be reproduced exactly. For sure, compression can be performed either directly with the (footprint of the) probe or with the fingertip (of the examiner’s contralateral hand) under real-time US guidance. Referred pain evoked by direct compression of the trigger point would be related to (i) the spatial orientation of the taut band harboring the nodule and (ii) the aforementioned biochemical milieu of the intercellular matrix activating/irritating the muscle nociceptors [4,5].

In the literature, Taheri et al. reported a sensitivity of 91% and specificity of 75% for US imaging to identify MTrPs within the upper fibers of the trapezius [19]. Likewise, Da Silva et al. found 100% agreement for intrarater reliability and 90% agreement for interrater reliability as regards gray-scale assessment of MTrPs in the upper trapezius [20].

### 3.3. Fascial Structures and Neural Elements

As previously mentioned, cervical MPS is a very complex clinical condition presenting with multiple pain generators cross-talking with each other. As such, not only the MTrPs but also the superficial and deep fasciae, peripheral nerves, and cutaneous branches of the dorsal rami (CBsDR) of spinal nerves can be involved in the clinical scenario [9].

***Superficial and deep fascia.*** The superficial fascia is a fibroelastic structure running within the subcutaneous tissue. It is connected to the overlying dermis and the underlying deep fascia through tiny skin ligaments known as the retinaculum cutis superficialis and profundus [21]. On the other hand, the deep fascia can be considered a multi-layer fibrous sheath connected to the surrounding muscles and tendons via myofascial expansions. The latter histologically and functionally connect multiple muscles to each other, generating the so-called myofascial chains [22]. For instance, the deep fascia of the trapezius muscle joins the fascia of the latissimus dorsi muscle caudally and the fascia of the serratus anterior and deltoid muscles laterally [23]. Spatial changes of chemical links connecting the hyaluronic acid chains located within the loose connective tissue between the fibrous layers of the deep fascia can lead to excessive stiffness. They are defined as fascial densification with intrafascial gliding impairment [24]. The deep portion of the subcutaneous fat is interposed between the superficial and deep fasciae, allowing differential gliding of the two layers. The two fascial structures mentioned above are richly innervated by free nerve endings (A δ and C nerve fibers) and play a pivotal role in the genesis of cervical myofascial pain [22]. Indeed, unlike the muscle tissue usually crossed by large nerve bundles, the fascial structures are widely invaded by a huge network of small nerve fibers, histologically defined as the fascial neural network [25].

***Peripheral nerves.*** The peripheral nerves most commonly involved in cervical MPS are the spinal accessory nerve (SAN) and the dorsal scapular nerve (DSN). Diffuse muscle spasm and/or bad posture can lead to chronic entrapment of these nerves, with progressive development of cervical neuropathic pain. Accordingly, several authors have proposed a US-guided block of the SAN [26] and/or DSN [27] to manage cervical myofascial pain refractory to first-line conservative treatments [23].

The SAN can be easily identified within the interfascial plane between the upper fibers of the trapezius and levator scapulae muscles (Figure 2). Before performing the US-guided block, the authors strongly recommend assessing the anatomical area with power Doppler imaging to promptly identify the superficial branch of the transverse cervical artery running within the same interfascial plane [28].

After piercing the medial scalene muscle, the DSN can be sonographically detected beneath the levator scapulae and rhomboid muscles, accompanied by the dorsal scapular artery (Figure 2) [29]. Of note, interscapular pain is quite often a referred pain originating from the neuro-myofascial tissues of the cervical spine. Herewith, the authors strongly suggest sonographically assessing the thoracic paraspinal and peri-scapular muscles to check for the eventual presence of muscle edema and/or injury at this level [30].

**Figure 3:**
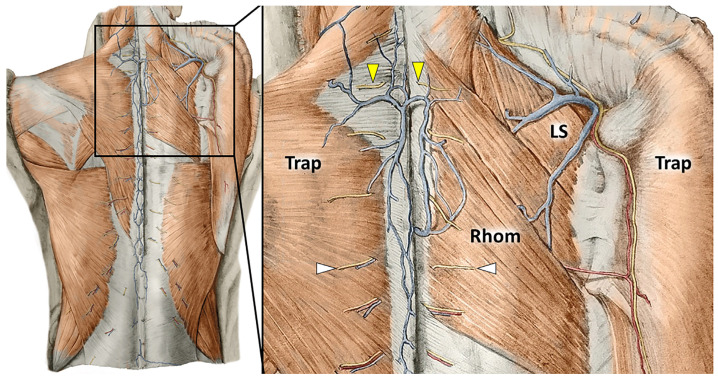
Anatomical drawings show the CBsDR of spinal nerves piercing the muscular (white arrowheads) and aponeurotic (yellow arrowheads) components of the trapezius (Trap) and rhomboid (Rhom) muscles to reach the subcutaneous tissue at the cervical/thoracic spine. LS: levator scapulae muscle.

***Cutaneous branches of the dorsal rami of the spinal nerves.*** Anatomical studies have described four main segments of the CBsDR of spinal nerves at the level of the cervical spine (Figure 3): intramuscular, epimuscular, intrafascial, and extrafascial portions [31,32].

Muscle contraction can surely compress the intramuscular segments of CBsDR. More interestingly, their intrafascial portion can also develop pathological entrapment, contributing to cervical neuropathic pain [33]. Indeed, mechanical forces transmitting along the deep fascia of the trapezius and/or rhomboid muscles can overstretch and irritate the tiny neural divisions of the CBsDR.

The clinical picture of cervical myofascial pain is quite often a complex melting pot of different pain generators interconnected with each other. For instance, multiple MTrPs of the trapezius muscle can lead to abnormal tension of its deep fascia, irritating the CBsDR of the spinal roots, which pierce the fascia at that level. Therefore, considering that each pain generator can influence the other one, it is quite hard to assign a specific label to the aforementioned painful condition, e.g., muscle pain, fascial pain, radicular pain.

### 3.4. US-Guided Procedures

In the pertinent literature, several types of manual therapies have been described to manage cervical myofascial pain. They are mainly based on two principles: (i) mechanical pressure over the MTrPs (e.g., the ischemic compression technique) and (ii) stretching of the affected muscle [34]. Considering the above-mentioned several pain generators that are potentially involved in cervical MPS, multiple US-guided interventions can be performed as well in clinical practice. In this regard, the authors suggest considering three main interventions: (i) injection and/or dry needling of the MTrPs, (ii) interfascial plane block, and (iii) fascial hydro-dissection [9,26,35].

A specific procedure or a combination of multiple interventions should be accurately selected in relation to both clinical and US findings [36]. For instance, US-guided dry needling of a single painful MTrP may not be the most suitable procedure in patients with diffuse contraction of the cervical muscles and potential entrapment of multiple CBsDR of spinal nerves. Fascial hydro-dissection or peripheral nerve block could be a more reasonable option for the latter. Needless to say, in challenging patients, interventions can also be performed for diagnostic purposes, i.e., to better identify the pain generator(s).

#### 3.4.1. Injection and/or Dry Needling of the MTrPs

Considering the above-quoted sonographic approach to MTrPs, it is crucial to accurately localize the hypoechoic nodule and its spatial relation with the surrounding neurovascular structures before performing the injection [9,10]. For example, deeply located cervical MTrPs can hardly be reached with the needle’s tip, and extra caution should be given as regards the closely surrounding vital structures, e.g., lung, pleura, and vertebral arteries [37]. Herein, the authors suggest considering alternative conservative treatments if the procedure appears to be too risky for an individual patient.

A linear, high-frequency US transducer is commonly used for imaging the cervical spine to ensure a high spatial resolution while assessing superficial tissues. The authors suggest using an in-plane technique to clearly visualize the needle for its whole length during the entire procedure [28]. Several approaches (e.g., lateral-to-medial or caudal-to-cranial) can be used, depending on the exact spatial location of the painful MTrP and the surrounding neurovascular structures. For instance, a medial-to-lateral technique can be used to inject a painful trigger point located within the rhomboid muscles in close proximity to the medial border of the scapula. Instead, a caudal-to-cranial approach can be used to target the upper fibers of the trapezius muscle. In the author’s experience, the upper fibers of the trapezius and the distal insertional zone of the levator scapulae muscles are the most frequent anatomical sites where MTrPs are clinically and sonographically identified.

As regards technical aspects of the procedure, also considering the aforementioned histopathological features of MTrPs, the authors suggest combining multiple steps and not only injecting inside the hypoechoic nodule [38]. However, if the local anesthetic can selectively target the nociceptors (A δ and C nerve fibers) of the trigger point, dry needling can promote the drainage of extra fluids entrapped within its intercellular matrix and bound to the glycosaminoglycans. Likewise, back-and-forward fan-like movements of the needle simultaneously allow for greater diffusion of the local anesthetic inside the pathological area and for mechanical disruption of the MTrP (Figure 1).

In the relevant literature, low-quality evidence suggests a superior effect of MTrP injections with local anesthetic for decreasing cervical muscle pain in the short term as compared with dry needling [39,40]. Based on systematic reviews and meta-analyses, the greater effect of the local anesthetic vs. dry needling suggests a pivotal role of peripheral sensitization in local and referred pain of cervical MPS [41,42]. Algogenic substances involved in increased responsiveness and reduced threshold of the A δ and C nerve fibers (i.e., peripheral sensitization) are histologically located inside the intercellular scaffold of the MTrP. Therefore, the authors once again highlight the synergistic effects of dry needling and anesthetic injection. The efficacy is mounted by the combination of the pharmacological effects of the anesthetic and the mechanical washout of the sensitizing substances.

#### 3.4.2. Interfascial Plane Block

In patients with diffuse cervical muscle contraction and absence of clinically and/or sonographically detectable painful MTrPs, US-guided injections of the interfascial planes can be performed to selectively block specific peripheral nerves. Indeed, within the virtual space between different muscular layers of the cervical spine, many neural structures run, and they are surrounded by connective tissue rich in collagen fibers and loose connective tissue of the areolar type with a variable number of adipose cells [23]. Among many, the SAN and the DSN are the most commonly involved nerves in cervical MPS [26,27].

The SAN can be easily visualized and blocked within the anatomical interface between the trapezius and levator scapulae muscles (Figure 2) [28]. The sonographic pattern similar to a “black ball” is related to the mono-fascicular architecture of the SAN at this level [43]. Positioning the US probe in the longitudinal axis, a cranial-to-caudal (or vice versa) approach can be performed to inject the intermuscular plane with the anesthetic agent, blocking the target nerve. In chronic peripheral nerve entrapments with fibrosis of the subsynovial connective tissue, perineural hydro-dissection using high volume improves kinematic properties of the entrapped nerve and its axoplasmic flow [44]. Accordingly, the authors suggest using high volumes rather than a few milliliters to “open” the interfascial plane. Lastly, taking into account the surrounding vascular structures, e.g., superficial and deep branches of the transverse cervical artery, power Doppler assessment in the pre-procedural phase should always be performed for a safer needle pathway (Figure 2).

The DSN can be promptly visualized and blocked within the anatomical plane between the rhomboid muscles and the thoracic wall (Figure 2) [25]. An in-plane technique with a medial-to-lateral approach is commonly preferred, since the DSN and the corresponding artery are in close proximity to the medial edge of the scapula. Unlike the SAN block, the DSN block is usually performed by more experienced physicians due to the presence of vital anatomical structures (e.g., lung, pleura) immediately deep into the target.

Interestingly, some authors have described a multi-target technique to manage myofascial upper back pain: (i) blocking the SAN and DSN through interfascial hydro-dissection, and (ii) injecting the trapezius, levator scapulae, and rhomboid muscles simultaneously [45]. Indeed, they speculated that the efficacy of nerve hydro-dissection can be strongly augmented by releasing the adjacent myofascial taut bands with a further decrease in the pressure on the entrapped nerves.

#### 3.4.3. Fascial Hydro-Dissection

**Figure 4:**
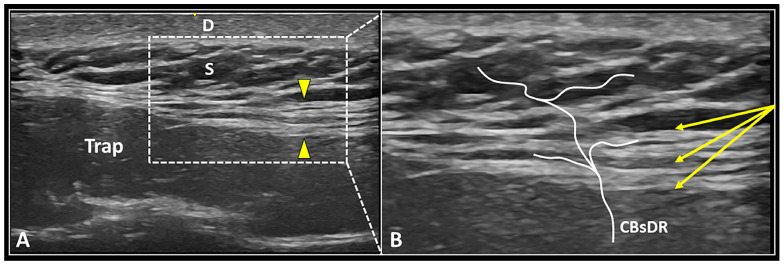
US image (**A**) shows the multi-layered deep fascia (yellow arrowheads) located between the upper fibers of the trapezius muscle (Trap) and the subcutaneous tissue (S). Under real-time US guidance (**B**), the needle (yellow arrows) can be advanced within the fascial structure, dissecting its different layers and releasing perforating cutaneous branches (CBsDR). D: dermis.

As previously mentioned, the deep fascia is a highly innervated and multi-layered fibrous structure that is extensively crossed by the CBsDR of spinal nerves [31,32]. Normally, the deep fascia presents a hyperechoic fibrillar echotexture (Figure 4), but in pathological conditions, hypoechogenicity, focal thickening, delamination, and perifascial effusion can be considered the most common sonographic findings [46,47].

More superficial layers of the deep fascia are in continuity with the hyperechoic honeycombing fibrous scaffold of the subcutaneous tissue, and they generate a unique functional framework [48]. In the pertinent literature, injections of the superficial soft tissues covering the paraspinal muscles have been widely described under the umbrella definition of mesotherapy or intradermal therapy [49]. Inhibitor spinal reflexes induced by needle insertion and intra- or subcutaneous release of the mixture are supposed to be the key mechanisms involved in the modulation of myofascial spinal pain [49,50].

US-guided hydro-dissection of the deep fascia can be considered an “advanced” interventional technique that simultaneously targets the intrafascial free nerve endings and the perforating cutaneous branches alike [25,31]. These tiny neural elements can concomitanly evoke superficial pain over the skin and deep pain mimicking muscle pain [51]. Moreover, layer-by-layer high-volume injection of the deep fascia can be considered a procedure that is potentially useful to restore the intrafascial gliding counteracting the above-quoted fascial densification [24,46]. As regards the technical tips and tricks, a long and flexible needle should be used to better “navigate” through the multiple layers of deep fascia. During both the advancement and retraction phases of the needle, the authors suggest slowly releasing the high-volume mixture to optimize the progressive detachment of the different layers of deep fascia—i.e., the seeding technique [35,48]. Last but not least, multiple back-and-forward movements of the needle within the deep fascia should be performed to better release the intrafascial adhesions in between its multiple layers.

## 4. Conclusions

Cervical MPS is a very common but quite challenging clinical condition. In this review, the authors proposed a ready-to-use guide in the clinical practice of musculoskeletal physicians. It is a pain generator-based approach to describing the different US-guided interventions while targeting the neuromyofascial cervical tissues.

## Figures and Tables

**Table 1:** Pain generator-based approach for US-guided procedures in cervical MPS.

Pain Generator	US-Guided Procedure	Tips and Tricks
MTrP	Injection and/or dry needling of the hypoechoic painful nodule	Multiple back/forward needle movements Fan-like technique
Peripheral Nerve (e.g., SAN, DSN)	Interfascial plane block	High-volume distension of the interfascial plane
Deep Fascia/CBsDR	Fascial hydro-dissection	Seeding technique to optimize layer-by-layer dissection

## Data Availability

Not applicable.
